# High-Yield Hydrogen Production from Starch and Water by a Synthetic Enzymatic Pathway

**DOI:** 10.1371/journal.pone.0000456

**Published:** 2007-05-23

**Authors:** Y.-H. Percival Zhang, Barbara R. Evans, Jonathan R. Mielenz, Robert C. Hopkins, Michael W.W. Adams

**Affiliations:** 1 Biological Systems Engineering Department, Virginia Tech, Blacksburg, Virginia, United States of America; 2 Chemical Sciences Division, Oak Ridge National Laboratory, Oak Ridge, Tennessee, United States of America; 3 Biosciences Division, Oak Ridge National Laboratory, Oak Ridge, Tennessee, United States of America; 4 Department of Biochemistry and Molecular Biology, University of Georgia, Athens, Georgia, United States of America; University of California, Berkeley, United States of America

## Abstract

**Background:**

The future hydrogen economy offers a compelling energy vision, but there are four main obstacles: hydrogen production, storage, and distribution, as well as fuel cells. Hydrogen production from inexpensive abundant renewable biomass can produce cheaper hydrogen, decrease reliance on fossil fuels, and achieve zero net greenhouse gas emissions, but current chemical and biological means suffer from low hydrogen yields and/or severe reaction conditions.

**Methodology/Principal Findings:**

Here we demonstrate a synthetic enzymatic pathway consisting of 13 enzymes for producing hydrogen from starch and water. The stoichiometric reaction is C_6_H_10_O_5_ (l)+7 H_2_O (l)→12 H_2_ (g)+6 CO_2_ (g). The overall process is spontaneous and unidirectional because of a negative Gibbs free energy and separation of the gaseous products with the aqueous reactants.

**Conclusions:**

Enzymatic hydrogen production from starch and water mediated by 13 enzymes occurred at 30°C as expected, and the hydrogen yields were much higher than the theoretical limit (4 H_2_/glucose) of anaerobic fermentations.

**Significance:**

The unique features, such as mild reaction conditions (30°C and atmospheric pressure), high hydrogen yields, likely low production costs ($∼2/kg H_2_), and a high energy-density carrier starch (14.8 H_2_-based mass%), provide great potential for mobile applications. With technology improvements and integration with fuel cells, this technology also solves the challenges associated with hydrogen storage, distribution, and infrastructure in the hydrogen economy.

## Introduction

Photosynthesis is the biological process that converts light energy to chemical energy and stores it in carbohydrates as “6 CO_2_ + 6 H_2_O→C_6_H_12_O_6_+6 O_2_”, and fixes atmospheric carbon into biomass (living carbon). Before the industrial revolution, the global economy was largely based on carbon extracted directly or indirectly (via animals) from plants; now the economy is mainly dependent on fossil fuels (dead carbon). At the dawn of the 21^st^ century, a combination of economic, technological, resource, and political developments is driving the emergence of a new carbohydrate economy [1], [2].

Climate change, mainly due to CO_2_ emissions from fossil fuel burning, and the eventual depletion of the world's fossil-fuel reserves, are threatening sustainable development [2]–[4]. Abundant, clean, and carbon-neutral hydrogen is widely believed to be the ultimate mobile energy carrier replacing gasoline, diesel, and ethanol; a high energy conversion efficiency (∼50–70%) can be achieved *via* fuel cells without producing pollutants [3]. Four main R&D priorities for the future hydrogen economy are: 1) decreasing hydrogen production costs *via* a number of means, 2) finding viable methods for high-density hydrogen storage, 3) establishing a safe and effective infrastructure for seamless delivery of hydrogen from production to storage to use, and 4) dramatically lowering the costs of fuel cells and improving their durability [5]–[7]. Hydrogen production from less costly abundant biomass is a shortcut for producing low-cost hydrogen without net carbon emissions [8]–[15].

Synthetic biology is interpreted as the engineering-driven building of increasingly complex biological entities for novel applications, involving the steps of standardization, decoupling, abstraction, and evolution [16]. One main goal of synthetic biology is to assemble interchangeable parts from natural biology into the systems that function unnaturally [17]. The simplest synthetic biology example is to assemble enzymes to implement an unnatural process, in which the gene regulatory systems do not exist. Here we apply the principles of synthetic biology to implement an important reaction by using 13 well-known enzymes, which form an unnatural enzymatic pathway. The most obvious advantage of this process is that the hydrogen yield is far higher than the theoretical yield (4 H_2_/glucose) of biological hydrogen fermentations [9], [15], [18]. This novel enzymatic high-yield hydrogen production method is anticipated to have great impacts on the future hydrogen and carbohydrate economy.

## Results

We designed a new enzymatic method for producing hydrogen from starch and water,(1)
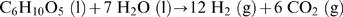



**Figure 1:**
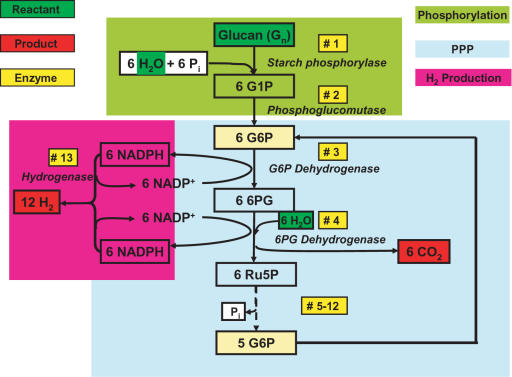
The synthetic metabolic pathway for conversion of polysaccharides and water to hydrogen and carbon dioxide. The abbreviations are: PPP, pentose phosphate pathway; G1P, glucose-1-phosphate; G6P, glucose-6-phosphate; 6PG, 6-phosphogluconate; Ru5P, ribulose-5-phosphate; and P_i_, inorganic phosphate. The enzymes are: #1, glucan phosphorylase; #2, phosphoglucomutase; #3, G-6-P dehydrogenase; #4, 6-phosphogluconate dehydrogenase, #5 Phosphoribose isomerase; #6, Ribulose 5-phosphate epimerase; #7, Transaldolase; #8, Transketolase, #9, Triose phosphate isomerase; #10, Aldolase, #11, Phosphoglucose isomerase: #12, Fructose-1, 6-bisphosphatase; and #13, Hydrogenase.

Figure 1 shows the synthetic enzymatic pathway that does not exist in nature. It is comprised of 13 reversible enzymatic reactions: a) a chain-shortening phosphorylation reaction catalyzed by starch phosphorylase yielding glucose-1-phosphate (Equation 2) [19]; b) the conversion of glucose-1-phosphate (G-1-P) to glucose-6-phosphate (G-6-P) catalyzed by phosphoglucomutase (Equation 3) [20]; c) a pentose phosphate pathway containing 10 enzymes (Equation 4) [21]; and d) hydrogen generation from NADPH catalyzed by hydrogenase (Equation 5) [22].(2)
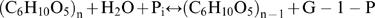

(3)
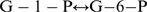

(4)
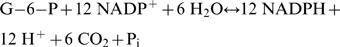

(5)
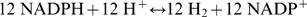



**Figure 2:**
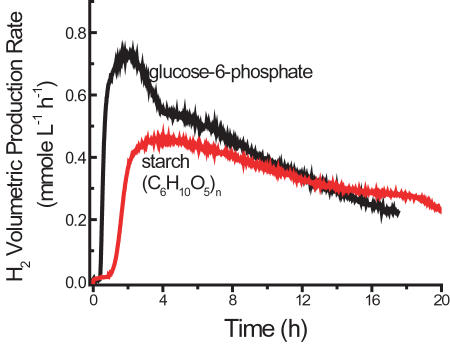
Hydrogen production from either 2 mM G-6-P or 2 mM starch (glucose equivalent). The reaction based on G-6-P contained the pentose phosphate cycle enzymes (#3-12, 1 unit each), ∼70 units of *P. furiosus* hydrogenase (#13), 0.5 mM thiamine pyrophosphate, 2 mM NADP^+^, 10 mM MgCl_2_, and 0.5 mM MnCl_2_ in 2.0 ml of 0.1 M HEPES buffer (pH 7.5), at 30°C. The reaction based on starch rather than G-6-P was supplemented by 10 units of α-glucan phosphorylase (#1), 10 units of phosphoglucomutase (#2), and 4 mM phosphate at 30°C.

**Figure 3:**
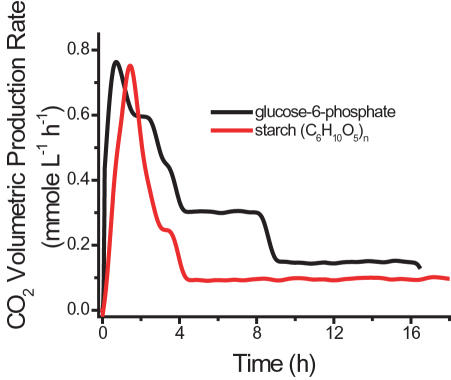
Carbon dioxide production from either 2 mM G-6-P or 2 mM starch (glucose equivalent). The experimental conditions were the same as those in Figure 2.

We first validated the reaction scheme of Woodward et al. [23], in which hydrogen was produced from G-6-P via 11 enzymes, based on the reaction of G-6-P+6 H_2_O→12 H_2_+6 CO_2_+P_i_ (top curve in Fig. 2). The proof-of-principle experiment was then conducted to validate whether hydrogen can be produced from starch and water at 30°C using 13 enzymes (see Materials and Methods). Clearly, hydrogen was produced as expected (bottom curve in Fig. 2). As compared to using G-6-P as the substrate, hydrogen production from starch exhibits a) a longer lag phase, b) a lower peak production rate (0.44 mmol/h/L), and c) an extended reaction time, all of which are consistent with the reaction mechanism (Fig. 1). The CO_2_ production for both cases was measured at the same time (Fig. 3). Clearly, CO_2_ was produced before H_2_ generation, which was in a good agreement with the mechanism in Figure 1. The integrated yields (mol/mol) of hydrogen and CO_2_, based on substrate consumption of G-6-P and starch, were 8.35 H_2_/G-6-P and 5.4 CO_2_/G-6-P, and 5.19 H_2_/glucose unit and 5.37 CO_2_/glucose unit, respectively. The yields of hydrogen and CO_2_ from G-6-P were approximately 70% and 86% of theoretical yields. The corresponding value for hydrogen from starch was lower (43%) although the CO_2_ yield was the same. The lower hydrogen yield was anticipated and its causes, such as the unfinished reaction, batch operation, and accumulation of metabolites (e.g., NADPH), are currently under study.

**Figure 4:**
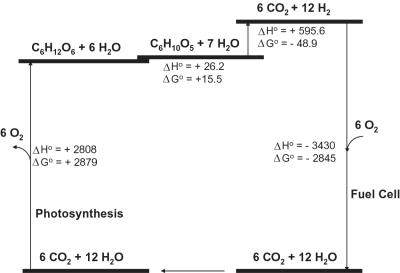
An energy diagram showing the standard enthalpy (Δ*H*°) and free energy changes (Δ*G*°) in kJ/mol for the reactions in a renewable energy cycle operating among H_2_O, CO_2_, glucose, and starch.

Thermodynamic analysis (Fig. 4) shows that the overall reaction (Equation 1) is a spontaneous process (i.e., ΔG° = −48.9 kJ/mol) and is a weakly endothermic reaction (i.e., ΔH° = 595.6 kJ/mol), based on data elsewhere [21], [24]. Since the gaseous products (H_2_ and CO_2_) are simultaneously removed from the liquid reaction solution, the real Gibbs free energy at 30°C and atmospheric pressure is much less than −48.9 kJ/mol, according to Le Chatelier's principle. The fairly large negative values of Gibbs free energy suggest a complete conversion. Sugar chain-shortening substrate phosphorylation (Eq. 2) utilizes the energy stored in the glucosidic bonds of polysaccharides (15.5 kJ/mol glucosidic bond) to produce the activated phosphorylated monosaccharide (G-1-P) without ATP consumption [20], [25] and avoids using expensive substrates such as glucose-6-phosphate [23]. The endothermic reaction suggests that some low-temperature heat energy from the environment is used to produce high quality energy carrier hydrogen, an extra 22% net energy gain. Although photosynthesis efficiency from solar energy to chemical energy is not so high as that of solar cells [26], hydrogen production based on inexpensive abundant biomass will be a shortcut to realization of the hydrogen economy without net carbon emissions, will avoid large capital investments for the hydrogen infrastructure, and will save the huge energy consumption currently required for production of solar cells [3].

## Discussion

There are four other means converting biomass to hydrogen: 1) direct polysaccharide gasification [8], [13]; 2) direct glucose chemical catalysis after polysaccharide hydrolysis [10], [11]; 3) anaerobic fermentations [9], [15], [18]; and 4) polysaccharide- or glucose-ethanol fermentations [27]–[29] followed by ethanol chemical reforming [12]. The chemical methods have low hydrogen yields (50∼57%) due to poor selectivity of catalysts and requires high reaction temperatures (e.g., 500∼900 K) [8], [10], [11], [13]. Anaerobic hydrogen fermentation is well known for its low hydrogen yield of 4 H_2_/glucose [9], [15], [18]. The combination of ethanol fermentation and ethanol-to-hydrogen reforming has a theoretical yield of 10 H_2_/glucose unit (e.g. 83% of the maximum). Allowing 5∼10% fermentation loss [30] and ∼5% reforming loss [12], the practical hydrogen yield through ethanol could be ca. 75% of the maximum yield. Assembly of the high-substrate-selectivity enzymes results in an artificial cascade enzymatic pathway, accompanied by a high hydrogen yield (12 H_2_/glucose), three time higher than the theoretical yield (4 H_2_/glucose) from biological hydrogen fermentations [9], [15], [18] and much higher than those from chemical catalysis [8], [10], [11].

Distinct from the severe reaction conditions of chemical catalysis [8], [10]–[14], the mild reaction conditions mediated by enzymes (∼20–100°C, depending on the enzymes employed) provide two obvious benefits: 1) easy implementation in a small space, especially for mobile applications, and 2) simple process configurations due to easy separation of the gaseous products (H_2_ and CO_2_) from the reactants (starch and water).

Costs of hydrogen production from less-costly starch (e.g., $∼0.15/kg) would be ∼$2/kg H_2_, assuming that feedstock costs account for half of overall costs and enzymes and co-enzyme account for another half. In general, approximately 40–75% of prices of commodities, such as gasoline from crude oil, hydrogen from natural gas, and ethanol from corn kernels, come from feedstock costs [31]. For example, current crude recombinant enzyme production costs are estimated to range ∼$10/kg; commercial cellulase production cost is as low as $1–2/kg [29]. Based on the rule of thumb for commodity production costs, the likely hydrogen-producing costs (∼$2/kg H_2_) could meet or exceed the hydrogen cost goals ($2–3/kg H_2_), established by the US DOE [32]. For example, the soaring prices of natural gas drove hydrogen costs from $1.40/kg H_2_ in 2003 to $2.70/kg H_2_ in 2005. We improve the method first described by Woodward [23] by starting with a less costly and abundant substrate–starch. Thus we avoid several major shortcomings of Woodward's method: 1) costly glucose-6-phosphate, 2) accumulation of phosphate, which is a strong inhibitor of fructose-1,6-bisphosphatase, 3) increasing ionic strength in the buffer, which slows down overall reaction rates, and 4) a pH shift in the buffer.

Solid starch has a relatively high energy density, with a mass-storage density of 14.8 H_2_-mass % and a volume-storage density of 104 kg H_2_/m^3^. These densities are higher than most of the solid hydrogen storage technologies [7], as well as exceeding the DOE goals of 4.5 mass%, 6 mass%, and 9 mass% in 2005, 2010, and 2015, respectively [5]. Replacement of conventional solid hydrogen storage technologies by the on-board starch-H_2_ converter and starch container will also solve several problems for solid hydrogen storage devices, e.g., energy loss for hydrogen compression or liquefaction, durability of reversible adsorption/desorption materials, high temperatures for desorption, and a long refilling time [5], [7]. Easy and safe storage and distribution of solid starch will address many issues of the hydrogen economy infrastructure. For example, setting up the infrastructure to store and distribute gaseous hydrogen to vehicles might cost hundreds of billions in the USA alone [33].

This robust synthetic enzymatic pathway that does not function in nature was assembled by 12 mesophilic enzymes from animal, plant, bacterial, and yeast sources, plus an archaeal hyperthermophilic hydrogenase. The performance (e.g., reaction rate and enzyme stability) is anticipated to be improved by several orders of magnitude by using the combination of (a) enzyme component optimization *via* metabolic engineering modeling [34], (b) interchangeable substitution of mesophilic enzymes by recombinant thermophilic or even hyperthermophilic enzymes [23], (c) protein engineering technologies, and (d) higher concentrations of enzymes and substrates. We have increased the hydrogen production rates by nearly 4 times greater than Woodward's results [23] through a) decreasing the ion strength of the buffer and b) substituting one mesophilic enzyme (#11). This research approach will naturally benefit from on-going improvements by others in synthetic biology systems that are addressing cofactor stability [35], enzyme stability by additives [36], and co-immobilization [37], and development of minimal microorganisms [38] that can be built upon to create an *in vivo* enzyme system that produces H_2_ in high yields.

The concept of cell-free synthetic enzymatic pathway engineering is anticipated to be applied to other commodity chemical production because of its unique benefits: high product yields (i.e., no formation of by-products and cell mass), modest reaction conditions as compared to chemical catalysis, no toxic chemicals required or produced, broad reaction conditions (e.g., high temperature and low pH) as compared with microorganisms, and easy operation and control. For example, it has been argued that cell-free ethanol fermentation systems would replace microbe-based ethanol fermentation someday [39].

With technology development and integration with PEM fuel cells, the starch-to-hydrogen conversion technology is anticipated to have wide mobile applications. We envision that future mobile appliances will store solid starch, produce hydrogen from starch and water via this reaction, and then generate electricity by hydrogen fuel cells at the same compact place.

## Materials and Methods

All chemicals and enzymes were purchased from Sigma Co, unless otherwise noted. All enzymes and their catalysis reactions are listed in Table 1.

**Table 1:** The enzymes used for hydrogen production from starch and water, and their reaction mechanisms, sources, and amounts used in the reaction.

E.C.	Enzyme Name	Reaction	Vender	Origin	Unit
2.4.1.1	glycogen phosphorylase	(C_6_H_10_O_5_)_n_+P_i_+H_2_O→(C_6_H_10_O_5_)_n−1_+glucose-1-P	Sigma	rabbit muscle	10
5.4.2.2	phosphoglucomutase	G-1-P→G-6-P	Sigma	rabbit muscle	10
1.1.1.49	glucose-6-phosphate dehydrogenase	G-6-P+NADP^+^→6-phosphogluconate+NADPH	Sigma	*S. cerevisiae*	1
1.1.1.44	6-phosphogluconic dehydrogenase	6-phosphogluconate+H_2_O+NADP^+^→ribulose-5-phosphate+NADPH+CO_2_	Sigma	*S. cerevisiae*	1
5.3.1.6	ribose 5-phosphate isomerase	ribulose-5-phosphate→ribose-5-phosphate	Sigma	spinach	1
5.1.3.1	ribulose-5-phosphate 3-epimerase	ribulose-5-phosphate→xylulose-5-phosphate	Sigma	*S. cerevisiae*	1
2.2.1.1	transketolase	xylulose-5-phosphate+ribose-5-phosphate→sedoheptulose-7-phosphate+glyceraldehyde-3-phosphate	Sigma	E. coli	1
		xylulose-5-phosphate+erythrose-4-phosphate→fructose-6-phosphate+glyceraldehyde-3-phosphate			
2.2.1.2	transaldolase	sedoheptulose-7-phosphate+glyceraldehyde-3-phosphate→fructose-6-phosphate+erythrose-4-phosphate	Sigma	*S. cerevisiae*	1
5.3.1.1	triose-phosphate isomerase	glyceraldehyde 3-phosphate→dihydroxacetone phosphate	Sigma	rabbit muscle	1
4.1.2.13	aldolase	glyceraldehyde 3-phosphate+dihydroxacetone phosphate→fructose-1,6-bisphosphate	Sigma	rabbit muscle	1
3.1.3.11	fructose-1,6-bisphosphate	fructose-1,6-bisphosphate+H_2_O→fructose-6-phosphate+Pi	[41]	*E. coli*	1
5.3.1.9	phosphoglucose Isomerase	fructose 6-phosphate→glucose-6-P	Sigma	*S. cerevisiae*	1
1.12.1.3	*P. furiosus* hydrogenase I	NADPH+H^+^→NADP^+^+H_2_	[22.42]	*P. furiosus*	∼70

**Figure 5:**
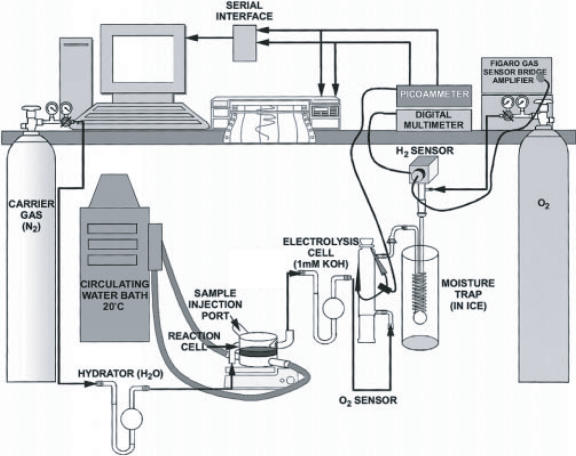
The hydrogen cell system configured for monitoring H_2_ with the ORNL in-house sensor based on the Figaro TGS 822 and O_2_ with a modified Hersh galvanic cell [43]. The CO_2_ analyzer (not shown) is attached between the reaction cell and the electrolysis cell.

The experiments were carried out in a continuous flow system as described previously [23], with the modification that the moisture traps were cooled with ice instead of liquid nitrogen, and that oxygen as well as hydrogen and carbon dioxide were monitored in the gas stream [23] (Fig. 5). The working volume of the custom reactor was 2 mL. The system was continuously purged with helium at a flow rate of 50 mL/min. The temperature of the jacketed reaction vessel was maintained at 30°C with a Polyscience (Niles, IL 60714) circulating water bath. Hydrogen evolution was measured with a Figaro TGS 822 tin oxide sensor connected over a bridge amplifier to a Keithley Model 2000 multimeter (Keithley Instruments, Cleveland, OH). Oxygen concentration was monitored with a modified Hersh galvanic cell using 24% KOH as the electrolyte connected to a Keithley autoranging picoammeter. Carbon dioxide production was measured with a LI-COR CO_2_ Analyzer Model LI-6252 connected to a Keithley 2000 multimeter. The multimeters and picoammeter were connected to a 486 computer through IEEE 488 general-purpose interface boards. Electrolysis for calibration of hydrogen and oxygen by Faraday's law of electrochemical equivalence was carried out with a Keithley 220 programmable current source connected to an in-line electrolysis cell. Calibration for carbon dioxide was carried out with an analyzed gas mixture consisting of 735 ppm carbon dioxide and 1000 ppm oxygen in helium (Air Liquide America Corp., Houston, TX 77056). Data collection and analysis was carried out with ASYST 4.0 software (ASYST Technologies, Inc., Rochester, NY).

The integrated molar/molar yields of hydrogen (Y_H2_) and carbon dioxide (Y_CO2_) are calculated as
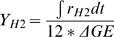


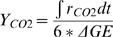
in which r_H2_ and r_CO2_ are the volumetric production rates in terms of mmole of H_2_ or CO_2_ per liter of reaction volume per hour, as shown in Figs. 2 and 3; Δ*GE*is the net consumption of glucose equivalent in terms of mM. Residual G-6-P can be measured using Sigma glucose HK kit [40]. The mixtures were incubated at 35°C for 5 minutes and the change in absorbance at 340 nm was determined. In the case of starch, the residual starch, G-1-P, and G-6-P were hydrolyzed to glucose by addition of dilute H_2_SO_4_ and hydrolysis at 121°C for 1 hour. The neutralized glucose solutions were measured by a glucose HK kit [40].
